# Rectosigmoid adenocarcinoma revealed by metastatic anal fistula. The visible part of the iceberg: a report of two cases with literature review

**DOI:** 10.1186/1477-7819-10-209

**Published:** 2012-10-03

**Authors:** El Bachir Benjelloun, Said Aitalalim, Leila Chbani, Ihsan Mellouki, Khalid Mazaz, Kahlid Aittaleb

**Affiliations:** 1 Department of Surgery, University hospital Hassan II, Fez, 30000, Morocco; 2 Department of Pathology, University hospital Hassan II, Fez, 30000, Morocco; 3 Department of Gastroenterology, University hospital Hassan II, Fez, 30000, Morocco

**Keywords:** Rectosigmoid cancer, Anal fistula, Implantation metastasis, Abdominoperineal resection

## Abstract

Colonic adenocarcinoma revealed by metastatic anorectal fistula is rare, with few cases in the literature. Such lesions can be taken for the more common manifestation of a benign perianal abscess or fistula. Once diagnosed, the management of such conditions remains controversial. We herein report two cases with perianal fistula that were subsequently found to have developed perianal adenocarcinoma on biopsy. Further colonic investigation revealed a rectosigmoid adenocarcinoma. Histology and immunohistochemical staining was identical in both primary and metastatic tumors. Preoperative chemoradiation with further rectal low anterior resection and local excision of metastatic anal fistula was performed. There is no recurrence after 3 years of follow-up.

## Background

Distal implantation by a proximal adenocarcinoma into an anal fistula remains a rare condition. It has been previously reported that exfoliated colorectal cancer cells can implant in distal locations such as the staple lines [1] and hemorrhoidectomy wounds [2] resulting in tumor recurrence. The same pathological mechanism can be expected in a case of colorectal adenocarcinoma seeding into anal fistula. Cancer implantation is usually suspected when carcinoma originating in an anal fistula has a similarity of histological findings between the primary colorectal cancer and the metastatic lesion [3]. Furthermore, the fistula must be present with a synchronous colorectal cancer. The diagnosis of such conditions remains difficult mainly when the primary tumor is unknown and the chronic anal fistula seems to be benign. More challenging is the management of such perineal tumors, as the literature is divided between surgeons who prefer radical abdominoperineal resection (APR) [4-17] and others who opt for sphincter-sparing surgery [18-26].

We herein present two cases of patients diagnosed with rectosigmoid adenocarcinoma revealed by metastatic perineal fistula, and treated with neoadjuvant chemotherapy followed by low anterior resection and local excision of the perineal tumor.

## Case 1

A 55-year-old man presented with a 10-year history of recurrent anal fistula. He had no history of inflammatory bowel disease. Physical examination revealed several external openings, in the right and left perianal regions. The internal opening was located posteriorly in proctoscopy. The patient underwent fistulectomy with seton placement in fistula tracts. The pathology of the fistula excision did not show any malignancy. After 3 months of follow-up, a discharging nodule appeared in the fistula tract wound. Rectal examination did not reveal any abnormalities in the anal canal or the rectum. The nodule was biopsied, and adenocarcinoma was found on histology review. Further colonic investigation revealed a rectosigmoid tumor.

**Figure 1:**
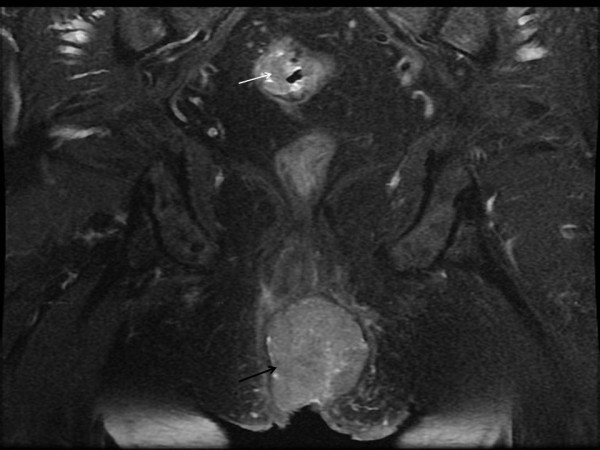
MRI of the pelvis showing the perineal mass (black arrow) and rectosigmoid tumor (white arrow).

**Figure 2:**
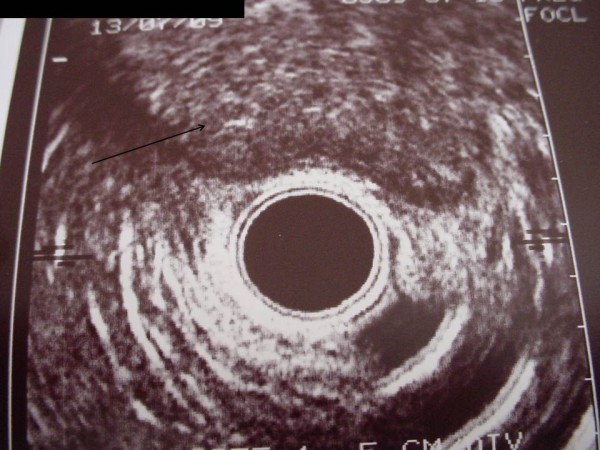
Endoanal ultrasound showing the perineal mass with sphincter involvement (black arrow).

Histology of both the primary tumor and the perianal nodule revealed a well-differentiated adenocarcinoma with cytokeratin 20 (CK20)+ and cytokeratin 7 (CK7)- staining. Magnetic resonance imaging (MRI) of the pelvis (Figure 1) and endoanal ultrasound (Figure 2) showed a perianal mass with anal sphincter involvement. Computer tomography (CT) did not show any distant metastasis. The two modalities of the treatment (including APR or neoadjuvant chemoradiation followed by low rectal resection and local excision of perineal mass with the risk of recurrence and incontinence) were discussed with the patient and informed consent was obtained. Eight weeks after a course of chemoradiation (45 Gy + 5 Fluorouracil), the patient underwent rectal anterior resection with colorectal anastomosis and local excision of the perianal mass which responded very well to the neoadjuvant therapy. Histopathological analysis of the excised nodule and rectosigmoid tumor revealed a well-differentiated adenocarcinoma as a primary tumor without lymph node involvement (T3N0M0), while the perianal mass showed a complete response following chemoradiotherapy. There is no recurrence after 3 years follow-up.

## Case 2

A 68-year-old man presented to the emergency room with a perianal abscess. He had no history of anal fistula. Examination under anesthesia was performed confirming the perianal abscess with an internal opening in the anal dentate line, proving the origin was a cryptoglandular fistula. Simple drainage was performed. Because the cavity of the abscess had an atypically indurated border, biopsy was done. Histology revealed a well-differentiated adenocarcinoma. Further investigation, including rectosigmoidoscopy, MRI of the pelvis and a CT scan, showed a large carcinoma 26 cm from the anal verge and a perianal mass extending to the anal sphincter, which was confirmed by endoanal ultrasound.

**Figure 3:**
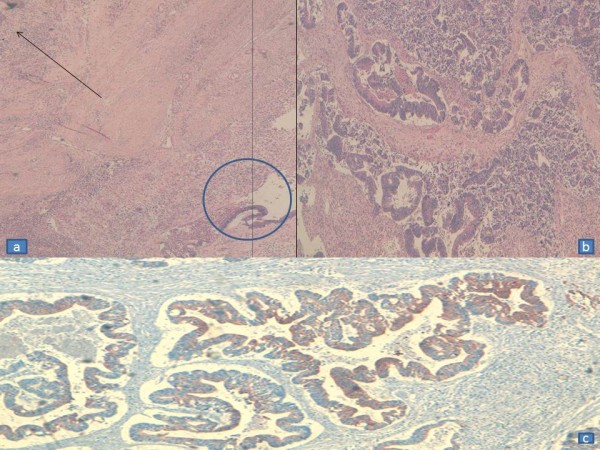
**Histology of the perineal fistula.a**) Glandular proliferation (circle) surmounted by a squamous epithelium (arrow). **b**) Adenocarcinoma with necrosis. **c**) Immunohistochemical staining immunoreactivity with anti-CK20.

No distant metastasis was noticed. Histology of the primary tumor was the same as the perianal mass. Immunohistochemistry for CK7 and CK20 was performed on tissues to distinguish colorectal adenocarcinoma from anal gland carcinoma. Both the colorectal cancer and the perianal tumor were CK7- and CK20+ (Figure 3). The modalities of the treatment (including APR or neoadjuvant chemoradiation followed by low rectal resection and local excision of the perineal mass with the risk of recurrence and incontinence) were discussed with the patient and informed consent was obtained. Preoperative chemoradiation (45 Gy + 5 Fluorouracil), was administered. Follow-up MRI 6 weeks after chemoradiation showed dramatic shrinkage of the perineal tumor. Surgery consisted of rectal anterior resection with colorectal anastomosis and local excision of the perianal mass. Pathological results of the rectosigmoid tumor revealed well-differentiated adenocarcinoma without lymph node involvement (T2N0M0). The perianal mass was completely sterilized. After 3 years follow-up there is no recurrence.

## Discussion

Cancer of the colon and rectum can be spread by direct extension, transperitoneal, lymphatic, and hematogenous spread. Spreading by implantation has been also described [2]. In 1907, Ryall reported that many solid cancers were implanted in the operative field, and described the phenomenona as ‘cancer infection’ [27]. Guiss reported the first case of cancer implantation into an anal fistula in 1954. Since then, a few other cases have been reported in the literature and the concept of cancer implantation has become widely accepted. We found 23 articles describing 24 patients with metastatic anal fistula arising from colorectal carcinoma [4-26]. Details of the cases are summarized in Table 1, along with the current patients. The diagnosis of such conditions remains difficult, mainly when the primary tumor is undiagnosed and the chronic anal fistula seems to be benign. It is, therefore the change in behavior of the anal fistula, such as the appearance of a nodule or perianal abscess with indurated borders as is described in our patients that should raise the red flag and awareness of possible malignancy, making biopsy and further colonic investigation mandatory. Histological differentiation and immunohistochemical staining must show the same characteristic in both tumors. More specifically, immunohistochemistry of CK7 and CK20 remains the examination of choice to distinguish between colonic tumor tissues and tumors originating from the anal glands [16]. Ramalingam *et al.* have previously described that anal glands were strongly immunoreactive to antibodies against CK7 but not to CK20 [28]. In our case, tumor cells of both lesions were positive for CK20 and negative for CK7 antibodies. We considered the tumor of the anal fistula as metastasis of the rectosigmoid cancer in both cases.

**Table 1:** **Reported cases of implantation metastasis from colorectal adenocarcinoma into a fistula*in ano***

**Author**	**Year**	**Age**	**Gender**	**Location of the primary tumor**	**Duration of anal fistula**	**Stage**	**Histology ADK Different**	**Operation**	**Follow up without recurrrence**
Guiss A [4]	1954	47	M	S	2 m	Dukes A	Mod	APR	1 y 2 m
Killingback *et al.*[5]	1965	63	M	S	8 y	Dukes A	Well	APR	NA
Parnes [6]	1976	47	M	S	3 m	Dukes B	Well	APR	18 m
Rollinson and Dundas [7]	1984	65	M	Rs	20 y	NA	Well	APR	10 m
Ueta *et al.*[8]	1991	66	F	S	44	Dukes B	Well	APR	6 m
Thomas and Thompson [9]	1992	68	M	S	1 y	Dukes B	Mod	APR	NA
Tohira *et al.* (Japenese) [10]	1998	75	M	UR	40 y	Dukes B	Well	APR	1 y
Isbister [18]	2000	47	M	Rs	2 y M	Dukes C	Mod	1- LR	NA
								2- LOR	
Isbister [18]	2000	39	M	S	N 1 y M	NA	NA	Refused APR	NA
Tokuhara *et al.*[11]	2001	69	M	S	5 y	Dukes B	Mod	APR	1 y
Yoshimura *et al.*[12]	2001	59	M	Rs	29 y	Dukes C	Mod	APR	3 y 7 m
Shinohara *et al.*[19]	2001	36	M	MR	16 y	Dukes C	Mod	AR + LR	6 m
Kouraklis *et al.*[13]	2002	75	M	S	1 y	Dukes B	Mod	APR	NA
Yagihashi *et al.*[14]	2002	50	M	S	NA	Dukes C	Well	TPE	3 y 8 m
Shimoyama *et al.*[15]	2003	61	M	Rs	5 y	Dukes C	Mod	APR	5 y
Hyman and Kida [16]	2003	66	M	S	15 y	Dukes B	Mod CK7-/CK20+	APR	1 y
Zbar and Shenoy [20]	2004	72	M	S	4 y	NA	NA	S + LR + adj CH RTH	1 y 2 m
Gupta *et al.*[21]	2005	44	M	Left colon	1 m	Dukes C	Mod	LH = LR	3 y
Hamada *et al.*[23]	2005	53	M	S	7 y	Dukes B	Well CK7-/CK20+	AR + LR	1 y
Ishiyama *et al.*[24]	2006	53	M	UR	20 y	Dukes C	Mod	AR + LR	10 m died from carcinomatosis
Sandiford *et al.*[25]	2006	72	M	Rs	2 y	Dukes B	Mod	S + LR + adj Ch R	14 m
Gravante *et al.*[17]	2008	64	M	Left colon	NA	Dukes A	Mod CK7-/CK20+	1-LH	1 y
								2-APR + adj CH RTH	
Wakatsuki *et al.*[26]	2008	57	M	Rs	7 y	Dukes B	Mod CK7-/CK20+	1-AR	3y7m
								After 2 years 2- LR	
Our report	2012	68	M	Rs	2 m	Dukes B	Well CK7-/CK20+	Neoadju chemoradia AR + LR	3 y
Our report	2012	55	M	Rs	10 y	Dukes B	Well CK7-/CK20+	Neoadju chemoradia AR + LR	3 y

S, sigmoid; Rs, rectosigmoid; R, rectum; Mod, moderately differentiated; Well, well-differentiated; APR, abdominoperineal resection; TPE, total pelvic exenteration; LAR, low anterior resection; LOR, local resection; adj, adjuvant; Neoadju chemoradia, neoadjuvant chemoradiation therpay; y, year(s); m, months; M, male; F, female; A, alive; L, left colon; LH, left hemicolectomy; UR, Upper rectum; MR, middle rectum; ADK, adenocarcinoma.CH: chemotherapy; RTH; radiotherapy; NA: not available.

Controversies remain in the management of metastatic anal fistula. It is difficult to define the best treatment from the literature, as some authors have performed APR, while others have chosen local resection.

After discussing the modality of treatment (either APR or neoadjuvant chemoradiation following by anterior rectal resection and local excision emphasizing the risk and the benefit of each approach, in particular risk of recurrence and incontinence) with our patients, we opted for preoperative chemoradiation (45 Gy), followed by anterior resection of the rectum and local excision of perianal metastasis after 6 weeks. Our choice was guided by the age of patients, differentiation and immunohistochemical characteristics of the primary and metastatic tumors, evidence of sphincter involvement on MRI and endoanal ultrasound and the absence of distant metastasis on abdominal CT. Furthermore, perineal masses in both cases shrank dramatically after chemoradiation. Consistent with that, the pathology report showed a complete response to chemoradiotherapy in both cases. The primary tumors were without lymph node metastases. Chemoradiation has been indicated as an adjuvant therapy in some previous reports [17,20,25], but not as a neoadjuvant treatment. The benefit of neoadjuvant versus adjuvant chemoradiation is still unclear. Based on a literature review of colorectal cancers implanting in a fistula track, patients undergoing local resection versus APR showed similar outcomes in terms of local recurrence. Nevertheless, it is difficult to make any concrete conclusion about the best approach, as the length of follow-up of reported cases is short.

## Conclusion

It is strongly recommended to be aware of any unusual changes in the clinical features of the fistula *in ano* either before or after surgery (such as increased discharge, bloody discharge, nodule formation, uncommonly slow healing or scar indurations) and not to hesitate to perform biopsy in order to exclude any malignancies that may be the visible part of the iceberg. Sphincter-saving surgery is an option that should be discussed with the patient.

## Consent

Written informed consent was obtained from the patients for publication of this case report and any accompanying images. A copy of the written consent is available for review by the Editor-in-Chief of this journal.

## Competing interests

The authors declare that they have no competing interests.

## Authors’ contributions

BEB has made substantial contributions to conception, bibliography and drafting the manuscript. AS has been involved in drafting the manuscript. CL has been involved in bibliography research and conception of pathology figures. IM and KM have been involved in or revising it critically for important intellectual content. KA has given final approval of the version to be published. All authors read and approved the final manuscript.
